# The Role of Extracellular Vesicles in the Progression of Human Neuroblastoma

**DOI:** 10.3390/ijms22083964

**Published:** 2021-04-12

**Authors:** Danilo Marimpietri, Irma Airoldi, Angelo Corso Faini, Fabio Malavasi, Fabio Morandi

**Affiliations:** 1 Laboratorio Cellule Staminali Post-Natali e Terapie Cellulari, IRCCS Istituto Giannina Gaslini, 16147 Genova, Italy; danilomarimpietri@gaslini.org (D.M.); irmaairoldi@gaslini.org (I.A.); 2 Dipartimento Scienze Mediche, Centro Ricerche Medicina Sperimentale (CeRMS) and Fondazione Ricerca Molinette Onlus, Università di Torino, 10121 Torino, Italy; angelo.faini@edu.unito.it (A.C.F.); fabio.malavasi@unito.it (F.M.)

**Keywords:** neuroblastoma, extracellular vesicles, tumor progression, immune modulation

## Abstract

The long-underestimated role of extracellular vesicles in cancer is now reconsidered worldwide by basic and clinical scientists, who recently highlighted novel and crucial activities of these moieties. Extracellular vesicles are now considered as king transporters of specific cargoes, including molecular components of parent cells, thus mediating a wide variety of cellular activities both in normal and neoplastic tissues. Here, we discuss the multifunctional activities and underlying mechanisms of extracellular vesicles in neuroblastoma, the most frequent common extra-cranial tumor in childhood. The ability of extracellular vesicles to cross-talk with different cells in the tumor microenvironment and to modulate an anti-tumor immune response, tumorigenesis, tumor growth, metastasis and drug resistance will be pinpointed in detail. The results obtained on the role of extracellular vesicles may represent a panel of suggestions potentially useful in practice, due to their involvement in the response to chemotherapy, and, moreover, their ability to predict resistance to standard therapies—all issues of clinical relevance.

## 1. Extracellular Vesicles: An Overview

The extracellular space of multi-cellular organisms contains solutions composed of metabolites, ions, proteins and polysaccharides, but also of a wide range of membrane-enclosed vesicles, henceforward referred as to “extracellular vesicles” (EV). EV are cell-derived sub-micronic vesicles released into the extracellular space in both normal and pathological conditions [1,2,3,4,5]. The importance of EV production in the living world has long been underestimated, with EV considered as irrelevant platelet residues or “cell dust” and initially discarded from studies [6]. EV are a highly conserved phenomenon, so much so that cells from all three domains of life, Archaea, Bacteria and Eukarya, produce small vesicles sometimes associated with filamentous structures, known as nanopods or nanotubes. Such ubiquity of the EV also suggests that their production may already have existed at the time of the last universal common ancestor (LUCA) [7].

EV comprise several subtypes that differ in size, morphology, composition (proteins, lipids, RNA, DNA) and biogenesis. Therefore, distinguishing one from another requires the use of specific methods of analysis for both isolation and characterization [8].

In this regard, the International Society for Extracellular Vesicles (ISEV) updated its Minimal Information for Studies of Extracellular Vesicles (MISEV) guidelines for the field in 2018, based on the evolution of the collective knowledge. The society specified that, in the absence of specific markers of reliable sub-cellular origin within the experimental systems, it was necessary to use operative terms for the subtypes of EV. These terms referred to the physical characteristics of the vesicles such as size (small EV and medium/large EV) or density (low, middle, high), in association with biochemical composition (CD9, CD63, CD81, ALIX, TSG101, HSPs, phosphatidylserine, Annexins) and/or description of conditions/cell origin “in the place of terms such as exosome and microvesicle that are historically burdened by both manifold, contradictory definitions and inaccurate expectations of unique biogenesis” [9].

Although the classification is continuously evolving, several recent authoritative articles [10,11,12] continued to use the traditional nomenclature, which generally classifies EV on the basis of their size and biogenesis. In 2019, for example, Jeppesen D.K. et al. [10] wrote a “reassessment of exosome composition” in which they described exosomes as endosome-derived small EV (40–150 nm). In terms of biogenesis, they specified that several molecules are actively and selectively incorporated into intraluminal vesicles (ILV), which reside within multivesicular bodies (MVB), and are then released into the extracellular space as exosomes, after fusion with plasma membrane. The authors improved the previous method for exosome purification, based on several steps of ultracentrifugation, by employing high-resolution iodixanol gradients (STAR methods) [10]. This allows for a more precise determination of exosome composition, revealing that classical tetraspanin-enriched exosomes contain a more limited repertoire of the diverse molecules present in the extracellular milieu than previously assumed. In the same study, microvesicles (MV) were reported as large EV (150–1000 nm) generated by the direct outward budding of the plasma membrane. In addition, they identified Annexin A1 as a specific marker for MV [10].

Later, in 2020, R. Kalluri and V.S. Le Bleu [11] divided EV into two major categories: exosomes (40–160 nm) and ectosomes, which include microvesicles, microparticles and large vesicles (50–1000 nm in diameter). Additionally, in 2020, K. O’Brien et al. [12] distinguished between four major subclasses of EV according to their different biogenesis pathways and size. They specifically referred to (i) exosomes (50–150 nm), (ii) MV (100–1000 nm), (iii) apoptotic bodies (100–5000 nm) and (iv) large oncosomes (1000–10,000 nm) containing abnormal and transforming macromolecules as well as other cargo [12].

These overlaps in size and other biophysical properties along with the lack of any unique markers make it difficult to define the different EV subclasses with confidence or to distinguish EV from aggregates such as lipoproteins that may be present in the same sample preparations.

EV exert different functional activities by transporting a variety of specific cargoes, including the genetic content of parent cells. EV can mediate cell communication in physiological tissue regulation, pathogenic injury and organ remodeling even at distant sites via biofluids. Interaction and uptake of these vesicles by recipient cells can be both selective and non-selective, depending on the specific molecules on their surfaces as well as their overall negative charge. In addition, a direct cell-to-cell transfer of extracellular vesicles through tunneling nanotubes was previously reported [12,13]. In this scenario, EV—and exosomes in particular—play a crucial role in tumorigenesis, tumor growth, metastasis and drug resistance. Tumor-derived EV are able to home in different tissues by virtue of the expression of specific integrins on their surface [14,15,16,17].

In addition, the detection and analysis of EV in pathological biofluids may provide a novel tool for observing cellular or tissue alterations for diagnostic readout. For this reason, the use of EV is arousing great interest as novel biomarkers for cancer diagnosis and prognosis prediction. In a previous publication, we [18] presented the first study on the isolation and characterization of human neuroblastoma (NB)-derived exosomes.

## 2. Characterization of Neuroblastoma-Derived EV

### 2.1. Neuroblastoma

NB is the most common pediatric extracranial solid tumor, with an incidence of roughly ten cases per million individuals [19]. NB arises from neural crest progenitor cells and is characterized by a peculiar biology and variable prognosis, ranging from spontaneous regression to highly malignant metastatic disease [19,20]. NB patients display different genetic features, including amplification of the proto-oncogene MYCN and recurrent segmental chromosome aberrations (losses of chromosome 1p, 3p, 4p, 11q and gains of 1q, 2p, 17q) [21,22]. An adverse prognostic factor is age at diagnosis, with a better event free survival (EFS) in young children than in older patients [23]. The combination of different clinical, biological and genetic parameters is used to stratify NB patients into different risk groups. Half of NB cases are high-risk (HR)-NB, with long-term survival rates below 40% despite multi-modality therapy [24], and present metastatic disease at diagnosis with less than 10% survival at relapse [25]. Patients presenting with metastatic spread (stage M) have a worse EFS than patients with localized tumors [20].

NB metastatic spread is mainly characterized by bone marrow (BM) and bone infiltration, with over 90% of stage 4 NB patients presenting with BM metastasis [26]. For this reason, BM is commonly used for molecular quantification of minimal residual disease and outcome prediction [27] and is extensively characterized as a peculiar metastatic site of NB [28]. Several studies showed that NB metastatic cells express distinctive features from primary tumor cells, including down-regulation of several tumor suppressor genes (i.e., SIRT6, BBC3/PUMA, STK11, CADM4 and GLTSCR2) and up-regulation of immunosuppressive molecules, such as calprotectin and HLA-G [29,30]. Moreover, the presence of metastatic NB cells can alter gene expression of BM resident cells, up-regulating genes which belong to innate immune response pathways [31]. In addition, different studies analyzed the transcriptomic landscape of NB with the aim of improving clinical prediction. Several genes implicated in neuronal differentiation were found to be over-expressed in low-risk tumors, whereas identification of novel prognostic markers for high-risk tumors or novel therapeutic targets is still a work in progress [32,33].

### 2.2. Liquid Biopsies

Different methodologies are used to examine the structure and features of invasive tumor lesions. In this respect, tissue biopsies have long been considered the gold standard for diagnostic purposes. However, with the era of precision medicine fast approaching, this approach has come under scrutiny and a number of limitations pointed out. These include the problem of acquiring an adequate amount of tissue, the limitation of reproducibility, the invasive nature of the method and tumor heterogeneity. Not least, tissue extraction may augment the risk of metastatic lesions in many cases [34].

In recent years, liquid biopsies are gaining interest as novel diagnostic molecular tools in oncology and as a source of biomarkers for cancer diagnosis, and the prognosis and determination of cancer predisposition. Mechanisms of resistance in refractory tumors may also be predicted by monitoring clonal evolution [35]. The main types of tumor-derived biological components in liquid biopsy include circulating tumor cells (CTC), circulating tumor DNA (ctDNA), tumor-derived RNA (e.g., miRNA), proteins and tumor-educated platelets (TEP) and EV [36,37]. Furthermore, the biological information contained in the liquid biopsy can reflect the comprehensive genome landscape deriving from multiple tumor sites. Compared with conventional tissue biopsy, liquid biopsy is becoming more useful because it is minimally- or non-invasive and entails lower costs in terms of time and money [38,39,40]. Liquid biopsy was also applied in the field of NB to improve diagnosis and therapy. Historically, NB is known to release CTC in the blood. Before the introduction of cancer liquid biopsies, their presence was used to serve as a circulating biomarker for treatment response [41]. Another potential circulating NB biomarker is tyrosine hydroxylase mRNA, which is consistently increased in NB patients and used to evaluate Minimal Residual Disease causing relapse, even after additional consolidation therapy [42]. Recent evidence demonstrates that several genomic alterations occurring in NB patients may be detected in ctDNA but not in primary tumor biopsies, indicating that liquid biopsy diagnostics may be better for highlighting tumor heterogeneity or detecting alterations present in metastases [43].

It 2as recently suggested that exosomes have several advantages over other biological components in liquid biopsies [44,45]. Indeed, among different moieties present in liquid biopsies, EV are stable in circulation and protect their cargos from degradation. In this context, we recently paved the way for using exosomal microRNA (exo-miRNA) in liquid biopsies as circulating biomarkers of chemotherapeutic response. The exo-miRNA expression profile in plasma samples collected from HR-NB children also provided a chemo-resistance index, which predicts good or poor prognosis [46], as further described below.

### 2.3. Presence of miRNAs

The presence of miRNAs in NB-derived EV was investigated by Haug and coworkers [47]. They isolated EV from Kelly and SK-NB-E NB cell lines, demonstrating that they contained both exosomes (as witnessed by the expression of tetraspanin CD9 and CD63, and of TSG101) and microvesicles. Moreover, NB-derived EV contained small RNA molecules, identified as miRNA. Analysis of these miRNA revealed that both cell lines expressed 11 miRNA (mir16, 125b, 21, 23a, 24, 25, 27b, 218, 320a, 320b and 92a), among which miR-92a was the highest expressed. However, using recipient cells transfected with miRNA sensing luciferase reporter vectors (specific for miR-92a, miR-9 and miR-21), the authors did not observe any effect after co-culture of such cells with NB-derived EV, suggesting that these miRNA may be not functional [47]. However, Challagundla and coworkers have demonstrated that miR-21 was expressed in exosomes isolated from different NB cell lines. Notably, miR-21 was functional, since it mediated the up-regulation of miR-155 in monocytes upon co-culture with NB cell lines [48].

### 2.4. Proteomic Analysis

A number of studies in the last years performed characterization of proteins contained in NB-derived EV. As mentioned above, we performed the first proteomic analysis on exosomes derived from NB cell lines [18]. The characterization of NB-derived EV was performed by size distribution and morphology analysis and revealed that they were enriched in exosomes. This conclusion was reinforced by the finding that these EV expressed tetraspanin molecules CD9, CD63 and CD81. The highest score in proteomic analysis performed on EV, derived from the HTLA-230 NB cell line (isolated from a patient with metastatic NB), was obtained for fibronectin and clathrin, which are exosome-associated proteins. Analysis of the cellular origin of proteins in NB-derived EV showed that the majority were derived from cytoplasm, whereas only a small percent of membrane-bound and nuclear proteins was detected. In addition, the involvement of such proteins in the biological process was characterized, showing that most of them pertain to metabolic processes, cell differentiation, cell proliferation, cell death and defense response. NB-derived EV were subjected to flow cytometric analysis and confirmed that some of these proteins, including fibronectin, the cancer stem cell marker CD133, the tumor associated antigen CD147 and the immunosuppressive molecule B7H3 (CD276), are expressed on their surface. Additionally, of note, strong expression of the disialoganglioside GD2, the most specific marker of NB, was detected in exosomes derived from all NB cell lines (HTLA-230, IMR-32, SH-SY5Y and GI-LIN), suggesting that plasma GD2+ EV could represent a novel potential biomarker for human NB [18].

An interesting study was conducted in 2017 by Colletti and coworkers [49] using the latest proteomic technology. The authors performed comparative proteomic analysis on EV derived from NB cell lines developed from primary tumors and BM metastasis. They identified 15 proteins exclusively present in EV derived from primary tumors. Among these proteins, all of which are involved in neuronal development, were (i) dystrophin, which is expressed in muscle and in brain, playing a role in synapse structure and function; (ii) TNFAIP3-interacting protein (TNIP1), which participates in repressing agonist-bound retinoic acid receptors and regulates the nuclear factor NF-kB pathway; (iii) ELAV-like neuronal protein 2 (ELAVL2), an RNA metabolism regulator related to neurodegenerative disorders; (iv) neuronal RNA-binding protein Nova-2 (NOVA2), which play a role in regulating the splicing of transcripts of proteins required for axonal pathfinding and (v) E3 ubiquitin-protein ligase pellino homolog 2 (PELI2), which helps regulate innate immunity signaling and is associated with neuronal inflammation [49]. In addition, primary tumor-derived EV showed expression of proteins involved in intra-Golgi traffic, autophagy and mitophagy, such as periodic tryptophan protein 1 homolog (PWP1), Rab GTPase-activating protein 1 (RABGAP1), TNFAIP3-interacting protein 1 (TNIP1) and calcium-binding and coiled-coil domain-containing protein 1 (CALCOCO1), which interact with GABA Type A Receptor Associated Protein Like 2 and 1 (GABARAPL2 and GABARAPL1) [49]. In contrast, proteins exclusively present in exosomes derived from BM metastatic cell lines were involved in cell survival, proliferation and cancer progression. These include (i) signal peptidase complex catalytic sub-unit SEC11 (SEC11A), which contributes to malignant progression of gastric cancer; (ii) cell division cycle-associated protein 3 (CDCA3) that is associated with cell cycle progression; (iii) nuclear pore complex protein Nup107 (NUP107) localized to kinetochores in mitosis, which is important for chromosome alignment and segregation and (iv) calcium and integrin-binding protein1 (CIB1), a protein implicated in cell survival, proliferation and angiogenesis, which is up-regulated in several cancers [49]. Nakata and coworkers [50] identified 92 proteins in common among EV derived from four different NB cell lines. These are several NB markers such as tyrosine 3-mono-oxygenase (Ty3H), secretogranin (SCG)-1 and -3 and chromogranin-A (CHGA), as well as proteins of neurosecretory granules. The majority of EV proteins derived from cytoplasm and were proteins typically found in exosomes, but DNA-binding proteins, extracellular matrix proteins and enzymes were also detected. In contrast, cytokines, growth factors and hormones were not enriched in EV preparations [50].

## 3. Release of EV: A Form of Bi-Directional Cross-Talk Between NB Cells and Other Cell Populations

Several studies over the last few years demonstrated that EV play an important role in NB, modulating tumor growth and invasiveness, and acting as an immune escape mechanism. In this context, EV released by NB cells can modulate the function of NB cells themselves and of other cell populations. In addition, a couple of studies demonstrated that EV released by cells of the immune system are captured by NB cells, thus blocking or increasing tumor cell growth.

Among all molecules contained in EV, miRNA are the most involved in modulating cell functions [48,51,52,53]. Gene transfer through EV release [54] and shedding of immunosuppressive molecules [55] were also described in NB. However, other molecules, including cytokines, mRNA, integrins, signal transduction proteins and ectoenzymes may also be involved in this phenomenon.

### 3.1. Delivery of NB-Derived EV to Mesenchymal Stem Cells

Two recent studies report that EV released by NB cells can modulate mesenchymal stem cell (MSC) functions.

Nakata and coworkers demonstrated that NB-derived EV are captured by MSC, and increase the secretion of different cytokines, such as VEGF, IL-17RA, IFN-α, IL-5, Il-6, IL-8, MCP-1 and MIP-1β, in the latter cells. Exosomes purified from these EV are also captured by MSC, where they increase the production of IL-6, IL-8, MCP-1 and VEGF, through ERK1/2 and AKT phosphorylation. Collectively, these data suggest that NB-derived EV (and purified exosomes) are capable of triggering a pro-tumorigenic phenotype in MSC, thus contributing to tumor spread [50]. The role of NB-derived EV in promoting tumor invasiveness and metastasis was also investigated by Colletti et al. [51]. They demonstrated that MSC isolated from the BM of NB patients with metastatic infiltration have a greater osteogenic potential than those isolated from patients without BM infiltration or healthy controls. Since osteoblast precursors stimulate osteoclast formation and bone resorption, the authors postulated that this feature of MSC may be associated with tumor progression. They investigated whether NB-derived EV are involved in the acquirement of osteogenic potential by MSC. First, they demonstrated that the uptake of EV derived from metastatic NB cell lines by MSC was higher than that detected with EV derived from primary tumor NB cell lines. More importantly, the uptake of EV from metastatic NB cell lines significantly up-regulated osteogenic mRNAs, such as BMP2, RUNX2, SPP1 and OSTERIX, in MSC. This effect was not seen in the presence of primary tumor NB cell line-derived EV [51]. The next step was to clarify the mechanism(s) involved in this process by analyzing a panel of miRNA in EV derived from metastatic or primary tumor NB cell lines. The expression of miR-375 was found to be higher in EV derived from metastatic NB cell lines than in those from primary tumor NB cell lines. Moreover, miR-375 expression was higher in MSC derived from NB-infiltrated BM than in those derived from non-infiltrated BM samples. miR-375 is able to regulate the expression of osteogenic genes through the suppression of two target genes: Yes-Associated Protein 1 (YAP1) and DEP domain containing mTOR-interacting protein (DEPTOR). The authors demonstrated that MSC cultured with EV derived from metastatic (but not primary tumor) NB cell lines showed an up-regulation of miR-375, thus confirming the role of NB-derived EV in the increase of the osteogenic potential of MSC, which is related to the metastatic spread of NB in the BM [51].

### 3.2. Delivery of EV to Other NB Cells

Other studies described the potential transfer of specific proteins through NB-derived EV that may confer a more aggressive phenotype to neighboring cells, thus supporting the invasiveness of the tumor. Fonseka and coworkers characterized EV derived from NB cell lines expressing N-Myc, which are more aggressive than N-Myc^−^ counterparts. The latter cells released fewer EV than N-Myc^+^ NB cell lines. Proteomic analysis of EV derived from these two cell populations revealed that both expressed markers of exosomes, such as Alix (PDCD6IP), TSG101, FlOT1 and VPS35. RAB proteins are differentially expressed in EV derived from N-Myc^+^ or N-Myc^−^ NB cell lines. EV derived from Myc^+^ NB cell lines expressed proteins involved in signal transduction, cell communication and transport (including IL3, mTOR, integrin and TRAIL signaling), whereas EV derived from N-Myc^−^ NB cell lines expressed protein involved in metabolism, cell growth and/or maintenance and regulation of nucleic acid metabolism. Interestingly, the authors demonstrated that N-Myc^−^ NB cell lines treated with EV derived from N-Myc^+^ NB cell lines display greater colony formation, migration and wound closure in vitro than untreated cells. In addition, N-Myc^−^ NB cell lines become resistant to chemotherapeutic agents in vitro. Such effects were abrogated when EV were isolated from N-Myc^+^ cells that underwent N-Myc knockdown, thus confirming the role of the transfer of N-Myc between these cell populations (through the release of EV) in the increase of aggressiveness [54].

Ma et al. [52] performed a next-generation sequencing on miRNA present in EV isolated from plasma of patients with NB, ganglioneuroblastoma (GNB) and healthy donors. Among the high number of miRNA that were up-regulated or down-regulated in NB/GNB patients, they identified only two miRNA that overlapped in miRNAs between NB/GNB patients and controls, and between NB and GNB patients, namely miR199a-3p and miR495-5p [52]. After validation by qRT-PCR on NB/GNB patients and healthy donors, only miR-199a-3p expression was confirmed to be higher in NB patients than in controls. In addition, miR-199-3p represents an independent risk factor, since its expression in the plasma was higher in NB patients with unfavorable histology than in those with a good prognosis [52]. Accordingly, the authors demonstrated a higher expression of miR-199-3p in NB cell lines and in NB cell line-derived exosomes than in non-NB cell lines (i.e., HUVEC). NB cell lines with low expression of miR-199-3p showed an increased proliferation and migration after transfection with the miR-199-3p mimic, and the same effect was observed after incubation with exosomes derived from other NB cell lines with high miR-199-3p expression. These data confirmed the role of miR-199-3p in the acquirement of aggressiveness by NB cell lines, and the role of EV as a vector [52]. Finally, transfection experiments revealed that NEDD4, a tumor suppressor gene, represents a target of miR-199-3p, since its expression was down-regulated in the presence of high amounts of miR-199-3p [52].

### 3.3. EV Released by Immune Cells May Modulate NB Cell Growth

Transfer of EV between NB cells and other cell populations is bi-directional and may influence immunosuppressive functions of NB cells and tumor growth. In this respect, an interesting study demonstrated that NB cells may be targeted by EV derived from tumor-infiltrating NK cells, leading to NB cell lysis [53]. First, the authors isolated EV from normal NK cell supernatants, demonstrating that such EV expressed tetraspanin CD81, TSG101, HSP70, the endosomal associated protein ALIX and the extracellular protein fibronectin. Next, they demonstrated that NB cell lines were lysed in vitro by IL-15 activated NK cells as well as by EV derived from the latter cells. Since those EV lack expression of common cytolytic molecules, such as perforin and Granzyme A and B, they hypothesized that other molecules, including miRNAs, may be involved. Among miRNAs contained in NK-derived EV, they identified miR-186-5p, since it targets important factors for NB survival, such as MYCN, AURKA and several components of the TGFb pathway [53]. They analyzed an RNA-seq data set from 498 primary NB samples, and they found that miR-186-5p was down-regulated in patients with high risk, N-myc amplified and stage 4 NB, compared to those with low risk, N-myc single copy and stage 1–3 NB. More importantly, patients with low miR-186-5p expression displayed a lower overall and event-free survival than those with normal miR-186-5p expression. The expression of NKG2D and DNAM-1 correlated with miR-186-5p expression in NB samples, thus suggesting that high expression of this miRNA is related to the presence of activated NK cells in the NB microenvironment. Moreover, TGF-β treatment significantly impaired miRNA-186-5p expression in NK cells and in their EV. The evidence that miR-186-5p is specifically delivered to NB cells by NK cell-derived EV (but not by EV derived from other cells) further confirmed that miR-186-5p is a tumor suppressor released by NK cells through EV, and that they may be suppressed by tumor cells as immune escape mechanism [53]. Finally, the authors demonstrated that delivery of miR-186-5p to NB cells down-regulated N-Myc, AURKA and TGFBR1/2, and dampened tumor cell proliferation and migration. Moreover, the delivery of miR-186-5p to tumor cells using anti-GD2-coated nanoparticles in a mouse model of NB significantly reduced tumor growth and prolonged survival of mice, thus suggesting a possible clinical application of this tumor suppressor [53].

In contrast, Challagundla et al. analyzed the effects of the transfer of miRNA between tumor cells and monocytes through EV release, demonstrating that such an interaction may increase tumor growth and invasiveness [48]. They found that miR-21 levels were increased in monocytes cultured in the presence of NB cell lines. Such an increase was abrogated when monocytes were cultured with supernatants depleted of exosomes. Moreover, miR-21 was up-regulated in monocytes transfected with RNA content of NB-derived EV, thus confirming that miR-21 is transferred from NB cells to monocytes through EV release [48]. The authors also demonstrated a bi-directional cross-talk between NB cells and monocytes, since miR-155 expression was up-regulated upon co-culture in both cell populations. Additionally, in this case, miR-155 was transferred between NB cells and monocytes through EV release. Transfer of miR-155 to NB cells leads to the down-regulation of the downstream gene TERF1, which is an inhibitor of telomerase activity. Thus, upon co-culture with monocytes, NB cells acquire aggressiveness and resistance to drugs. Indeed, NB cells treated with Dotap-miR-155 showed an increased growth and telomere length as compared to NB cells treated with Dotap-Scrambled in the presence of CDDP (election drug for the treatment of NB patients). Such drug resistance, induced by miR-155, can be reverted by up-regulation of TERF1 [48]. NB cells are able to induce M2 polarization of unpolarized monocytes, as witnessed by the up-regulation of M2 markers and CD163. Moreover, upon co-culture, miR-21 and miR-155 were up-regulated in both M1 and M2 polarized monocytes, whereas TERF1 expression was increased in NB cells. These data were also validated in vivo, since tumors with high TAM infiltration displayed a higher expression of miR-155 and a lower expression of TERF1. Preclinical studies revealed that mice injected with a co-culture of NB cells and monocytes and then treated with peri-tumoral injection of Dotap-miR-155 displayed a higher tumor growth as compared to mice treated with Dotap-Scrambled. In addition, mice injected with NB cell lines transfected with anti-miR-21 (in combination with monocytes and MSC) display a lower number of CD163+ cells and a down-regulation of miR-155 and miR-21, as compared with mice inoculated with NB cells transfected with an anti-scrambled sequence. Collectively, these data demonstrated a bi-directional transfer of miRNA between NB cells and monocytes, which leads to a polarization of TAM to M2 phenotype and to an increased aggressiveness and drug resistance in tumor cells [48].

## 4. Immunosuppressive Functions of NB-Derived EV

EV released by a tumor may be endowed with immunosuppressive molecules derived by parental cells, thus contributing to the establishment of a tolerogenic microenvironment by stimulating the function of MDSC and regulatory T cells and, on the other hand, by inhibiting anti-tumor activities of T lymphocytes and NK cells. Suppressive molecules contained in tumor-derived EV or expressed on EV membrane included cytokines and chemokines, ligands for inhibitory receptors (i.e., PVR, PD-L1, FasL, MIC A/B), miRNAs, HLA-G and adenosinergic ectoenzymes [56,57,58,59].

We previously reported in multiple myeloma the presence of ectoenzymes belonging to two different adenosinergic pathways for the production of adenosine (ADO). The canonical pathway is started by CD39, which converts ATP to AMP, whereas the alternative pathway hinges on CD38, which converts NAD to ADPR. The latter molecule is the substrate of CD203a/PC-1 which generate AMP. Both pathways converge on CD73, which converts AMP to ADO [60]. These ectoenzymes are expressed by different cell populations in the myeloma niche, where both pathways are functional, leading to the production of ADO [61]. We demonstrated that EV released by myeloma cells contributed to the increase of ADO in the myeloma niche, which in turn favors the progression of the disease by the establishment of an immunosuppressive microenvironment [61,62].

We recently characterized the immunosuppressive function of NB-derived EV. In particular, we analyzed the expression and function of adenosinergic ectoenzymes CD38, CD39, CD73 and CD203a/PC-1 on NB cell lines, metastatic NB cells derived from patients’ BM samples, and on NB-derived MV. First, we demonstrated that NB cell lines expressed higher levels of CD38 and CD39, and lower levels of CD73 and CD203a/PC-1, than metastatic NB cells. Similar results were obtained by analyzing MV isolated from these two NB cell populations, with the exception of CD39, whose expression was higher in MV from metastatic NB than in those from NB cell lines [55]. We also demonstrated that adenosinergic ectoenzymes are expressed also on resident BM cell populations (lymphocytes, granulocytes and monocytes). Such expression was not affected by the presence of metastatic NB cells in the BM microenvironment, with the exception of CD73, which was up-regulated on myeloid cells upon NB infiltration. Indeed, we demonstrated that the expression of CD73 (and also that of CD38 and CD39) increased upon co-culture with NB cells in vitro. Thus, these data suggested that MV endowed with adenosinergic ectoenzymes may be released in the BM microenvironment by both resident cells and infiltrating NB cells [55]. Next, we characterized EV isolated from BM plasma samples of NB patients and healthy controls. NB-derived EV expressed higher levels of CD38, CD39, CD73 and CD203a/PC-1, in terms of antigen density and percentage of positive EV. Moreover, the percentage of EV with high expression of CD38 and CD203a/PC-1 was higher in those from NB than in those from controls. Accordingly, the enzymatic function of NB derived EV was higher than those isolated from controls. In particular, the activity of CD39 (witnessed by the conversion of ATP to ADP and AMP) and of CD73 (demonstrated by the ability to convert AMP to ADO) was higher in MV from NB patients than in those from controls, whereas the enzymatic functions of CD203a/PC-1 and CD38 were similar in these two groups [55]. Finally, we highlighted the immunosuppressive functions of NB-derived EV, since these EV were able to impair T cell proliferation in vitro. Interestingly, the presence of EV expressing CD38, CD39 and CD73 (but not CD203a/PC-1) in the BM correlated with a worse event-free survival of NB patients, suggesting that NB-derived EV may dampen anti-tumor immune response in vivo. Such immunosuppressive function is, at least in part, related to the activity of the “classical” adenosinergic pathway (which hinges on CD39/CD73 enzymatic function) on those EV, that leads to local production of the immunosuppressive molecule, ADO [55].

The ability of NB-derived EV to dampen tumor-specific T cell responses was also addressed by Ali and coworkers [63]. They characterized NB-derived EV, demonstrating the expression of CD81 tetraspanin, syntenin and TSG101 cytosolic proteins on the EV preparation. Moreover, they demonstrated that such EV are able to suppress the cytotoxic activity of CD171 CAR T cells against NB cell lines in vitro. This effect was limited to CD4+ CAR T cells, whereas CD8+ CAR T cells were unaffected. The release of IFN-γ by CAR T cells was only partially impaired by NB-derived EV, whereas IL-2 secretion was unaffected. More importantly, T cell viability was not altered by priming of CAR T cells with NB-derived EV. Taken together, these data suggested that NB-derived EV are involved in the modulation of anti-tumor T cell response, and such immunosuppressive activity must be taken into account in the design of immunotherapeutic strategies for NB patients [63].

## 5. EV Released by NB May Predict Response to Chemotherapy

As mentioned above, we recently described a comparative analysis of EV derived from peripheral blood collected from high-risk NB patients [46]. Patients were all at INSS stage 3–4, and 50% of them displayed N-Myc amplification. The patients were first divided into two groups on the basis of response to chemotherapy (minor response, MR or very good partial response, VGPR) and prognosis (good or poor, considering relapse, tumor progression and overall survival) [46]. EV preparations were enriched in exosomes, as witnessed by CD9 expression. GD2 expression was detected in 50% of exosomes isolated from patients at diagnosis, and in 27% of exosomes isolated from patients after treatment, thus suggesting that induction chemotherapy reduced tumor-derived EV in the peripheral blood of patients. Next, the miRNA profile of total EV-RNA isolated from patients was evaluated at diagnosis and after induction chemotherapy. A total of 62 miRNA were found to be down-regulated, suggesting they had a role in the response to treatment. Interestingly, only three miRNA, namely, miR-29c, miR-342-3p and let-7b, were downregulated in MR but not in VGPR NB patients. These data were validated in RT-qPCR, thus confirming the role of these miRNA in the response to treatment. Indeed, pathway analysis confirmed that miR-29c, miR-342-3p and let-7b are involved in pathways that play a major role in cancer development and chemoresistance, particularly in focal adhesion, p53, FoxO and PI3K-Akt signaling pathways. The final step was to analyze the following miRNAs, recognized in the literature to be modulated by different drugs used for treating NB patients: cisplatin (CDDP), carboplatin (CBDCA), etoposide (VP-16), doxorubicin (DOXO) and vincristine (VCR). A chemoresistance index (CI) towards each drug was calculated for each patient by dividing the number of EV-associated miRNA modulated by the total number of miRNAs associated with a response to the particular drug. Examination of CI values and INSS stage, response to chemotherapy, MYCN status or EFS revealed two main clusters, and only EFS was found to be significantly associated with both. In particular, cluster 1 was associated with poor responders and showed (i) high CI toward CBDCA, CDDP and DOXO, and (ii) low CI toward VP-16 and VCR. In contrast, cluster 2 displayed the opposite CI values and was associated with good responders. In conclusion, the study demonstrated that NB-derived EV are modulated by chemotherapy, and, more importantly, that the miRNA content of EV may predict chemoresistance in NB patients [46].

## 6. Conclusions

**Figure 1:**
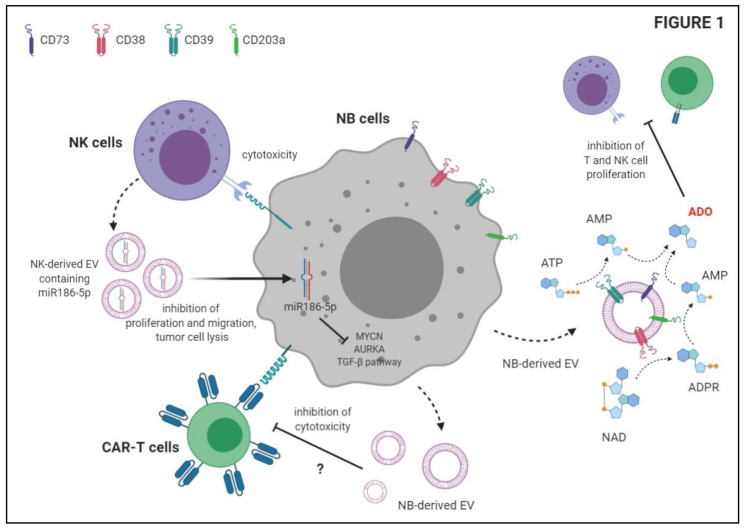
Schematic depiction of the immunosuppressive functions of Neuroblastoma-derived extracellular vesicles (EV) and anti-tumor activities of NK-derived EV.

Recent studies described EV release by NB cells as a mechanism that abrogates anti-tumor immune response. As summarized in Figure 1, NB-derived EV may promote a tolerogenic microenvironment in primary tumors as well as in metastatic sites by increasing the concentration of immunosuppressive molecules (i.e., ADO). This type of EV may directly inhibit the anti-tumor activities of CAR T cells and possibly of other immune cell populations as well. Instead, EV released by immune cells infiltrating the tumor may be captured by NB cells and abrogate tumor growth.

**Figure 2:**
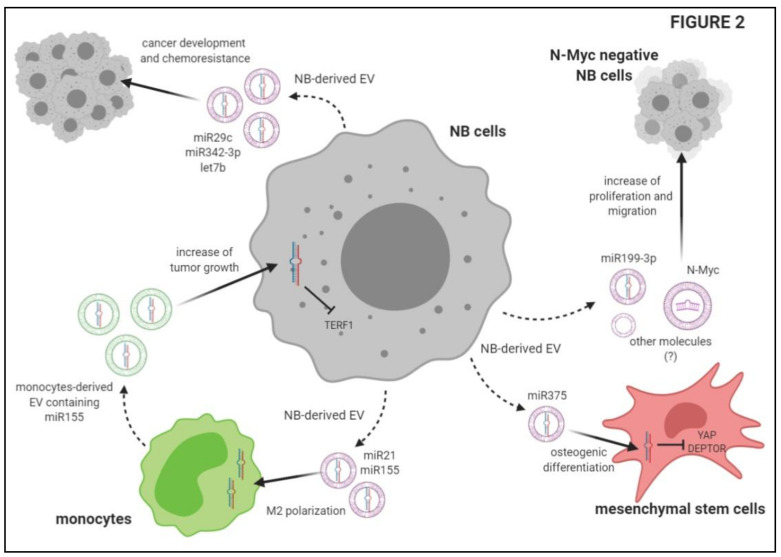
Schematic description of the mechanisms underlying the effects of extracellular vesicles on tumor growth and metastatic spread of Neuroblastoma cells.

In addition, as summarized in Figure 2, EV released by NB cells are able to promote tumor growth and metastases. This is achieved by the transfer of miRNAs or genes from NB cells to neighboring NB cells, or by modulation of the function of other cell populations present in the tumor microenvironment, such as MSC and monocytes. In this context, EV released by the latter cells are able to increase tumor growth. Finally, EV released by NB cells may be modulated by chemotherapy, and their composition may predict resistance to standard therapies.

Given the important role of EV in NB progression and in the control of anti-tumor immune response, it is tempting to speculate that drugs which selectively target EV may be used in combination with conventional therapies to improve clinical outcomes of high-risk NB patients. Indeed, different pathways may be targeted to block EV cross-talk, starting from EV biogenesis and release. Different proteins are involved in this process, including Endosomal Sorting Complex Required for Transport (ESCRT); neutral sphingomyelinase (n-SMase), an enzyme central to ceramide production; Rab27 family and dimethyl amiloride (DMA), an inhibitor of Na^+^/H^+^ and Na^+^/Ca^2+^ exchangers. It was previously demonstrated that inhibition of such components may have beneficial effects on cancer treatments [64,65,66]. Another approach is related to the removal of EV from the circulation, using specific antibodies for tumor-associated antigens expressed by tumor-derived EV or lectins and a plasmapheresis platform [67]. Finally, uptake of EV by target cells, which is generally mediated by phosphatidylserine exposed on the EV surface or heparan sulfate proteoglycans (HSPGs) present on recipient cells, may be blocked by Annexin V (or its homodimer Diannexin) or heparin, respectively. These drugs were successfully administered in preclinical models of squamous carcinoma and glioblastoma [64,66].

Other recent findings suggest that it may be possible to manipulate, ex-vivo, exosomes derived from parental tumors or from dendritic cells and MSC, which can be loaded with drugs, proteins, miRNA, DNA and RNA, and infused as bio-carriers to deliver these molecules to the tumor [59]. Indeed, EV may be a promising therapeutic tool since they act as intercellular messengers, are considered non immunogenic and are able to protect their cargoes from serum proteases and the immune system, avoiding phagocytosis or degradation. Due to their immunomodulatory capacity and their ability to home to tumor sites, MSC may be a reliable source of EV for this purpose. Nevertheless, to enhance the therapeutic potential of naïve EV, researchers developed several methods to load exogenous molecules into these vesicles, involving passive and active cargo loading. These data may support the therapeutic strategy of using the MSC-derived EV as a next-generation drug delivery platform [68].

In conclusion, EV represent a key player in the development and spread of NB. Their characterization and evaluation must be taken into account in the design of novel therapeutic strategies for children with high-risk NB. On the other hand, NB-derived EV may also themselves represent a potential novel therapeutic target for these NB patients.

## References

[1] György B., Szabó T.G., Pásztói M., Pál Z., Misják P., Aradi B., László V., Pállinger E., Pap E., Kittel A. (2011). Membrane vesicles, current state-of-the-art: Emerging role of extracellular vesicles. Cell. Mol. Life Sci. CMLS.

[2] Lane R.E., Korbie D., Hill M.M., Trau M. (2018). Extracellular vesicles as circulating cancer biomarkers: Opportunities and challenges. Clin. Transl. Med..

[3] Théry C., Ostrowski M., Segura E. (2009). Membrane vesicles as conveyors of immune responses. Nat. Rev. Immunol..

[4] Yáñez-Mó M., Siljander P.R.M., Andreu Z., Zavec A.B., Borràs F.E., Buzas E.I., Buzas K., Casal E., Cappello F., Carvalho J. (2015). Biological properties of extracellular vesicles and their physiological functions. J. Extracell. Vesicles.

[5] Teng F., Fussenegger M. (2020). Shedding Light on Extracellular Vesicle Biogenesis and Bioengineering. Adv. Sci..

[6] Wolf P. (1967). The nature and significance of platelet products in human plasma. Br. J. Haematol..

[7] Gill S., Catchpole R., Forterre P. (2019). Extracellular membrane vesicles in the three domains of life and beyond. FEMS Microbiol. Rev..

[8] Van Niel G., D’Angelo G., Raposo G. (2018). Shedding light on the cell biology of extracellular vesicles. Nat. Rev. Mol. Cell Biol..

[9] Thery C., Witwer K.W., Aikawa E., Alcaraz M.J., Anderson J.D., Andriantsitohaina R., Antoniou A., Arab T., Archer F., Atkin-Smith G.K. (2018). Minimal information for studies of extracellular vesicles 2018 (MISEV2018): A position statement of the International Society for Extracellular Vesicles and update of the MISEV2014 guidelines. J. Extracell. Vesicles.

[10] Jeppesen D.K., Fenix A.M., Franklin J.L., Higginbotham J.N., Zhang Q., Zimmerman L.J., Liebler D.C., Ping J., Liu Q., Evans R. (2019). Reassessment of Exosome Composition. Cell.

[11] Kalluri R., LeBleu V.S. (2020). The biology, function, and biomedical applications of exosomes. Science.

[12] O’Brien K., Breyne K., Ughetto S., Laurent L.C., Breakefield X.O. (2020). RNA delivery by extracellular vesicles in mammalian cells and its applications. Nat. Rev. Mol. Cell Biol..

[13] Rustom A., Saffrich R., Markovic I., Walther P., Gerdes H.-H. (2004). Nanotubular Highways for Intercellular Organelle Transport. Science.

[14] Siveen K.S., Raza A., Ahmed E.I., Khan A.Q., Prabhu K.S., Kuttikrishnan S., Mateo J.M., Zayed H., Rasul K., Azizi F. (2019). The Role of Extracellular Vesicles as Modulators of the Tumor Microenvironment, Metastasis and Drug Resistance in Colorectal Cancer. Cancers (Basel).

[15] Hoshino A., Costa-Silva B., Shen T.-L., Rodrigues G., Hashimoto A., Tesic Mark M., Molina H., Kohsaka S., Di Giannatale A., Ceder S. (2015). Tumour exosome integrins determine organotropic metastasis. Nature.

[16] Melo S.A., Sugimoto H., O’Connell J.T., Kato N., Villanueva A., Vidal A., Qiu L., Vitkin E., Perelman L.T., Melo C.A. (2014). Cancer Exosomes Perform Cell-Independent MicroRNA Biogenesis and Promote Tumorigenesis. Cancer Cell.

[17] Beltraminelli T., Perez C.R., De Palma M. (2021). Disentangling the complexity of tumor-derived extracellular vesicles. Cell Rep..

[18] Marimpietri D., Petretto A., Raffaghello L., Pezzolo A., Gagliani C., Tacchetti C., Mauri P., Melioli G., Pistoia V. (2013). Proteome profiling of neuroblastoma-derived exosomes reveal the expression of proteins potentially involved in tumor progression. PLoS ONE.

[19] Maris J.M. (2010). Recent advances in neuroblastoma. N. Engl. J. Med..

[20] Cohn S.L., Pearson A.D., London W.B., Monclair T., Ambros P.F., Brodeur G.M., Faldum A., Hero B., Iehara T., Machin D. (2009). The International Neuroblastoma Risk Group (INRG) classification system: An INRG Task Force report. J. Clin. Oncol. Off. J. Am. Soc. Clin. Oncol..

[21] Pinto N.R., Applebaum M.A., Volchenboum S.L., Matthay K.K., London W.B., Ambros P.F., Nakagawara A., Berthold F., Schleiermacher G., Park J.R. (2015). Advances in Risk Classification and Treatment Strategies for Neuroblastoma. J. Clin. Oncol. Off. J. Am. Soc. Clin. Oncol..

[22] Matthay K.K., Maris J.M., Schleiermacher G., Nakagawara A., Mackall C.L., Diller L., Weiss W.A. (2016). Neuroblastoma. Nat. Rev. Dis. Primers.

[23] London W.B., Boni L., Simon T., Berthold F., Twist C., Schmidt M.L., Castleberry R.P., Matthay K.K., Cohn S.L., De Bernardi B. (2005). The role of age in neuroblastoma risk stratification: The German, Italian, and children’s oncology group perspectives. Cancer Lett..

[24] Louis C.U., Shohet J.M. (2015). Neuroblastoma: Molecular pathogenesis and therapy. Annu. Rev. Med..

[25] London W.B., Castel V., Monclair T., Ambros P.F., Pearson A.D.J., Cohn S.L., Berthold F., Nakagawara A., Ladenstein R.L., Iehara T. (2011). Clinical and Biologic Features Predictive of Survival After Relapse of Neuroblastoma: A Report From the International Neuroblastoma Risk Group Project. J. Clin. Oncol..

[26] Mehes G., Luegmayr A., Ambros I.M., Ladenstein R., Ambros P.F. (2001). Combined automatic immunological and molecular cytogenetic analysis allows exact identification and quantification of tumor cells in the bone marrow. Clin. Cancer Res. Off. J. Am. Assoc. Cancer Res..

[27] Burchill S.A., Beiske K., Shimada H., Ambros P.F., Seeger R., Tytgat G.A., Brock P.R., Haber M., Park J.R., Berthold F. (2017). Recommendations for the standardization of bone marrow disease assessment and reporting in children with neuroblastoma on behalf of the International Neuroblastoma Response Criteria Bone Marrow Working Group. Cancer.

[28] Morandi F., Corrias M.V., Pistoia V. (2015). Evaluation of bone marrow as a metastatic site of human neuroblastoma. Ann. N. Y. Acad. Sci..

[29] Morandi F., Scaruffi P., Gallo F., Stigliani S., Moretti S., Bonassi S., Gambini C., Mazzocco K., Fardin P., Haupt R. (2012). Bone marrow-infiltrating human neuroblastoma cells express high levels of calprotectin and HLA-G proteins. PLoS ONE.

[30] Rifatbegovic F., Frech C., Abbasi M.R., Taschner-Mandl S., Weiss T., Schmidt W.M., Schmidt I., Ladenstein R., Ambros I.M., Ambros P.F. (2018). Neuroblastoma cells undergo transcriptomic alterations upon dissemination into the bone marrow and subsequent tumor progression. Int. J. Cancer.

[31] Scaruffi P., Morandi F., Gallo F., Stigliani S., Parodi S., Moretti S., Bonassi S., Fardin P., Garaventa A., Zanazzo G. (2012). Bone marrow of neuroblastoma patients shows downregulation of CXCL12 expression and presence of IFN signature. Pediatric Blood Cancer.

[32] Vermeulen J., De Preter K., Naranjo A., Vercruysse L., Van Roy N., Hellemans J., Swerts K., Bravo S., Scaruffi P., Tonini G.P. (2009). Predicting outcomes for children with neuroblastoma using a multigene-expression signature: A retrospective SIOPEN/COG/GPOH study. Lancet Oncol..

[33] Oberthuer A., Hero B., Berthold F., Juraeva D., Faldum A., Kahlert Y., Asgharzadeh S., Seeger R., Scaruffi P., Tonini G.P. (2010). Prognostic impact of gene expression-based classification for neuroblastoma. J. Clin. Oncol. Off. J. Am. Soc. Clin. Oncol..

[34] Robertson E.G., Baxter G. (2011). Tumour seeding following percutaneous needle biopsy: The real story!. Clin. Radiol..

[35] Alix-Panabieres C., Pantel K. (2016). Clinical Applications of Circulating Tumor Cells and Circulating Tumor DNA as Liquid Biopsy. Cancer Discov..

[36] Bellassai N., D’Agata R., Jungbluth V., Spoto G. (2019). Surface Plasmon Resonance for Biomarker Detection: Advances in Non-invasive Cancer Diagnosis. Front. Chem..

[37] In’t Veld S., Wurdinger T. (2019). Tumor-educated platelets. Blood.

[38] Siravegna G., Marsoni S., Siena S., Bardelli A. (2017). Integrating liquid biopsies into the management of cancer. Nat. Rev. Clin. Oncol..

[39] Alix-Panabières C., Pantel K. (2021). Liquid Biopsy: From Discovery to Clinical Application. Cancer Discov..

[40] Ignatiadis M., Sledge G.W., Jeffrey S.S. (2021). Liquid biopsy enters the clinic—Implementation issues and future challenges. Nat. Rev. Clin. Oncol..

[41] Seeger R.C., Reynolds C.P., Gallego R., Stram D.O., Gerbing R.B., Matthay K.K. (2000). Quantitative Tumor Cell Content of Bone Marrow and Blood as a Predictor of Outcome in Stage IV Neuroblastoma: A Children’s Cancer Group Study. J. Clin. Oncol..

[42] Uemura S., Ishida T., Thwin K.K.M., Yamamoto N., Tamura A., Kishimoto K., Hasegawa D., Kosaka Y., Nino N., Lin K.S. (2019). Dynamics of Minimal Residual Disease in Neuroblastoma Patients. Front. Oncol..

[43] Van Paemel R., Vlug R., De Preter K., Van Roy N., Speleman F., Willems L., Lammens T., Laureys G., Schleiermacher G., Tytgat G.A.M. (2020). The pitfalls and promise of liquid biopsies for diagnosing and treating solid tumors in children: A review. Eur. J. Pediatr..

[44] Li S., Yi M., Dong B., Tan X., Luo S., Wu K. (2020). The role of exosomes in liquid biopsy for cancer diagnosis and prognosis prediction. Int. J. Cancer.

[45] Yu W., Hurley J., Roberts D., Chakrabortty S.K., Enderle D., Noerholm M., Breakefield X.O., Skog J.K. (2021). Exosome-based liquid biopsies in cancer: Opportunities and challenges. Ann. Oncol..

[46] Morini M., Cangelosi D., Segalerba D., Marimpietri D., Raggi F., Castellano A., Fruci D., de Mora J.F., Cañete A., Yáñez Y. (2019). Exosomal microRNAs from Longitudinal Liquid Biopsies for the Prediction of Response to Induction Chemotherapy in High-Risk Neuroblastoma Patients: A Proof of Concept SIOPEN Study. Cancers.

[47] Haug B.H., Hald Ø H., Utnes P., Roth S.A., Løkke C., Flægstad T., Einvik C. (2015). Exosome-like Extracellular Vesicles from MYCN-amplified Neuroblastoma Cells Contain Oncogenic miRNAs. Anticancer Res..

[48] Challagundla K.B., Wise P.M., Neviani P., Chava H., Murtadha M., Xu T., Kennedy R., Ivan C., Zhang X., Vannini I. (2015). Exosome-mediated transfer of microRNAs within the tumor microenvironment and neuroblastoma resistance to chemotherapy. J. Natl. Cancer Inst..

[49] Colletti M., Petretto A., Galardi A., Di Paolo V., Tomao L., Lavarello C., Inglese E., Bruschi M., Lopez A.A., Pascucci L. (2017). Proteomic Analysis of Neuroblastoma-Derived Exosomes: New Insights into a Metastatic Signature. Proteomics.

[50] Nakata R., Shimada H., Fernandez G.E., Fanter R., Fabbri M., Malvar J., Zimmermann P., DeClerck Y.A. (2017). Contribution of neuroblastoma-derived exosomes to the production of pro-tumorigenic signals by bone marrow mesenchymal stromal cells. J. Extracell. Vesicles.

[51] Colletti M., Tomao L., Galardi A., Paolini A., Di Paolo V., De Stefanis C., Mascio P., Nazio F., Petrini S., Castellano A. (2020). Neuroblastoma-secreted exosomes carrying miR-375 promote osteogenic differentiation of bone-marrow mesenchymal stromal cells. J. Extracell. Vesicles.

[52] Ma J., Xu M., Yin M., Hong J., Chen H., Gao Y., Xie C., Shen N., Gu S., Mo X. (2019). Exosomal hsa-miR199a-3p Promotes Proliferation and Migration in Neuroblastoma. Front Oncol..

[53] Neviani P., Wise P.M., Murtadha M., Liu C.W., Wu C.H., Jong A.Y., Seeger R.C., Fabbri M. (2019). Natural Killer-Derived Exosomal miR-186 Inhibits Neuroblastoma Growth and Immune Escape Mechanisms. Cancer Res..

[54] Fonseka P., Liem M., Ozcitti C., Adda C.G., Ang C.S., Mathivanan S. (2019). Exosomes from N-Myc amplified neuroblastoma cells induce migration and confer chemoresistance to non-N-Myc amplified cells: Implications of intra-tumour heterogeneity. J. Extracell. Vesicles.

[55] Morandi F., Marimpietri D., Horenstein A.L., Corrias M.V., Malavasi F. (2019). Microvesicles expressing adenosinergic ectoenzymes and their potential role in modulating bone marrow infiltration by neuroblastoma cells. Oncoimmunology.

[56] Raimondo S., Pucci M., Alessandro R., Fontana S. (2020). Extracellular Vesicles and Tumor-Immune Escape: Biological Functions and Clinical Perspectives. Int. J. Mol. Sci..

[57] Czernek L., Düchler M. (2017). Functions of Cancer-Derived Extracellular Vesicles in Immunosuppression. Arch. Immunol. Ther. Exp. (Warsz).

[58] Rebmann V., König L., Nardi Fda S., Wagner B., Manvailer L.F., Horn P.A. (2016). The Potential of HLA-G-Bearing Extracellular Vesicles as a Future Element in HLA-G Immune Biology. Front. Immunol..

[59] Dutta A. (2020). Exosomes-based cell-free cancer therapy: A novel strategy for targeted therapy. Immunol. Med..

[60] Ferretti E., Horenstein A.L., Canzonetta C., Costa F., Morandi F. (2019). Canonical and non-canonical adenosinergic pathways. Immunol. Lett..

[61] Horenstein A.L., Quarona V., Toscani D., Costa F., Chillemi A., Pistoia V., Giuliani N., Malavasi F. (2016). Adenosine Generated in the Bone Marrow Niche Through a CD38-Mediated Pathway Correlates with Progression of Human Myeloma. Mol. Med..

[62] Morandi F., Marimpietri D., Horenstein A.L., Bolzoni M., Toscani D., Costa F., Castella B., Faini A.C., Massaia M., Pistoia V. (2018). Microvesicles released from multiple myeloma cells are equipped with ectoenzymes belonging to canonical and non-canonical adenosinergic pathways and produce adenosine from ATP and NAD+. Oncoimmunology.

[63] Ali S., Toews K., Schwiebert S., Klaus A., Winkler A., Grunewald L., Oevermann L., Deubzer H.E., Tüns A., Jensen M.C. (2020). Tumor-Derived Extracellular Vesicles Impair CD171-Specific CD4(+) CAR T Cell Efficacy. Front. Immunol..

[64] El Andaloussi S., Mäger I., Breakefield X.O., Wood M.J.A. (2013). Extracellular vesicles: Biology and emerging therapeutic opportunities. Nat. Rev. Drug Discov..

[65] Kosaka N., Yoshioka Y., Fujita Y., Ochiya T. (2016). Versatile roles of extracellular vesicles in cancer. J. Clin. Invest..

[66] Vader P., Breakefield X.O., Wood M.J.A. (2014). Extracellular vesicles: Emerging targets for cancer therapy. Trends Mol. Med..

[67] Marleau A.M., Chen C.S., Joyce J.A., Tullis R.H. (2012). Exosome removal as a therapeutic adjuvant in cancer. J. Transl. Med..

[68] Baek G., Choi H., Kim Y., Lee H.-C., Choi C. (2019). Mesenchymal Stem Cell-Derived Extracellular Vesicles as Therapeutics and as a Drug Delivery Platform. Stem Cells Transl. Med..

